# Supplementary material for: Broadband giant-refractive-index material based on mesoscopic space-filling curves
Source: Nat Commun. 2016 Aug 30;7:12661. doi: 10.1038/ncomms12661 (PMC5013611; doi:10.1038/ncomms12661)
Supplement: Supplementary Information — Supplementary Figures 1-12 and Supplementary Notes 1-4 [file ncomms12661-s1.pdf]

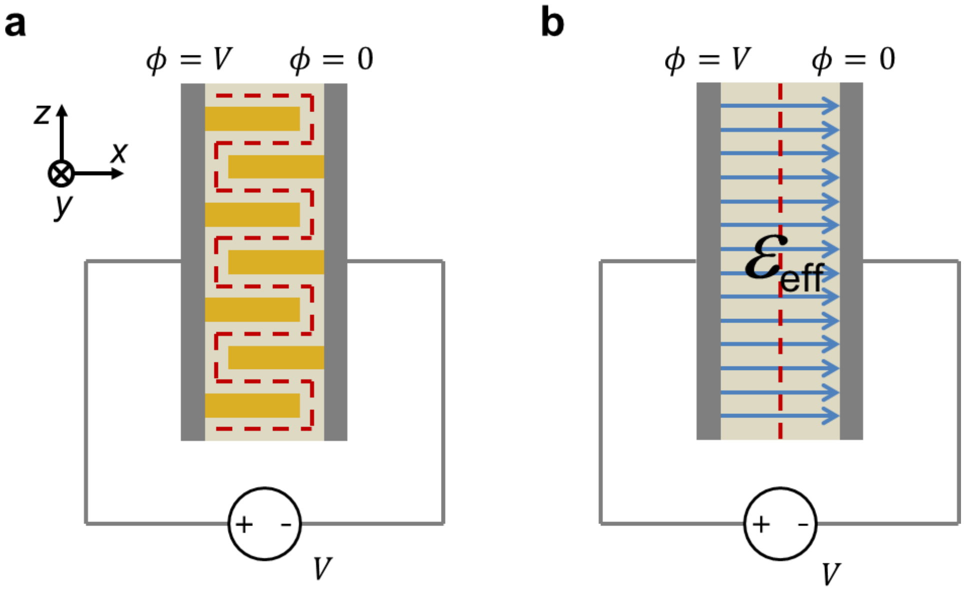

Supplementary Figure 1 | Find the effective dielectric constant from the capacitance (a) Put the proposed structure into a parallel plate capacitor where voltage, V, is applied. (b) Equivalent situation of (a). The homogenized dielectric material gives same capacitance in (a). In (a) and (b), red dashed line indicate the equipotential line of $V/2$.

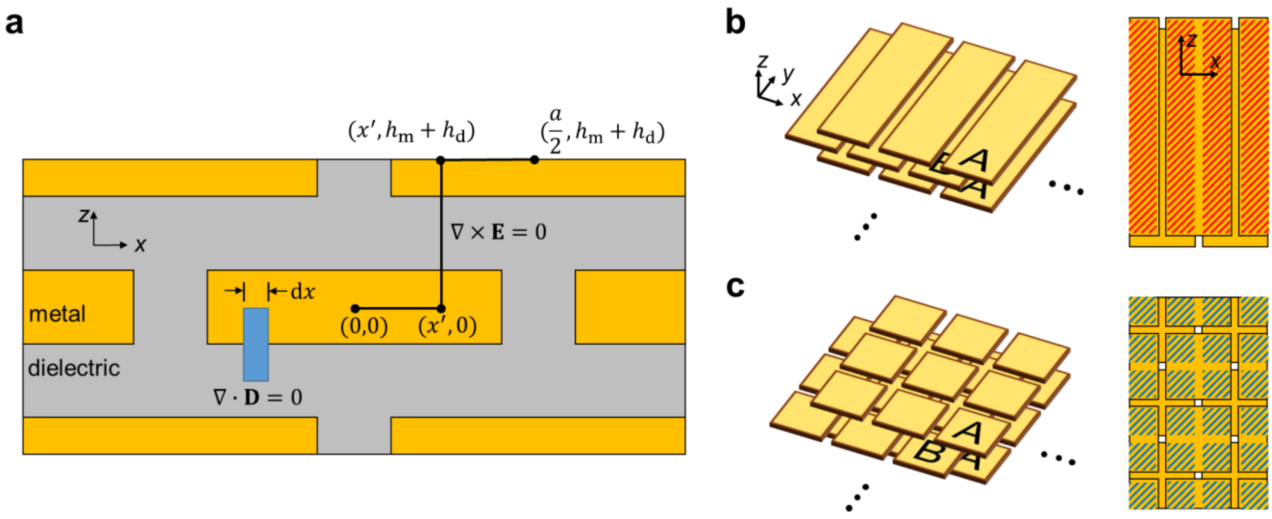

Supplementary Figure 2 | The schematic of the proposed mesoscopic crystal (a) Cross section view of the mesoscopic crystal. Yellow and gray color indicate the metal strip and host dielectric, respectively. Structural dimensions are same as in main text. Within quasi-static regime, integration of electric field along black solid line is constant for any x' and integration of electric displacement flux along blue surface is zero. (b) A schematic and a top view of biaxial mesoscopic crystal. Red dashes indicate overlap region between metal plates. (c) Similar one for the uniaxial mesoscopic crystal (alternatively stacked in both x and y direction). Blue dashes indicate overlap region between metal plates.

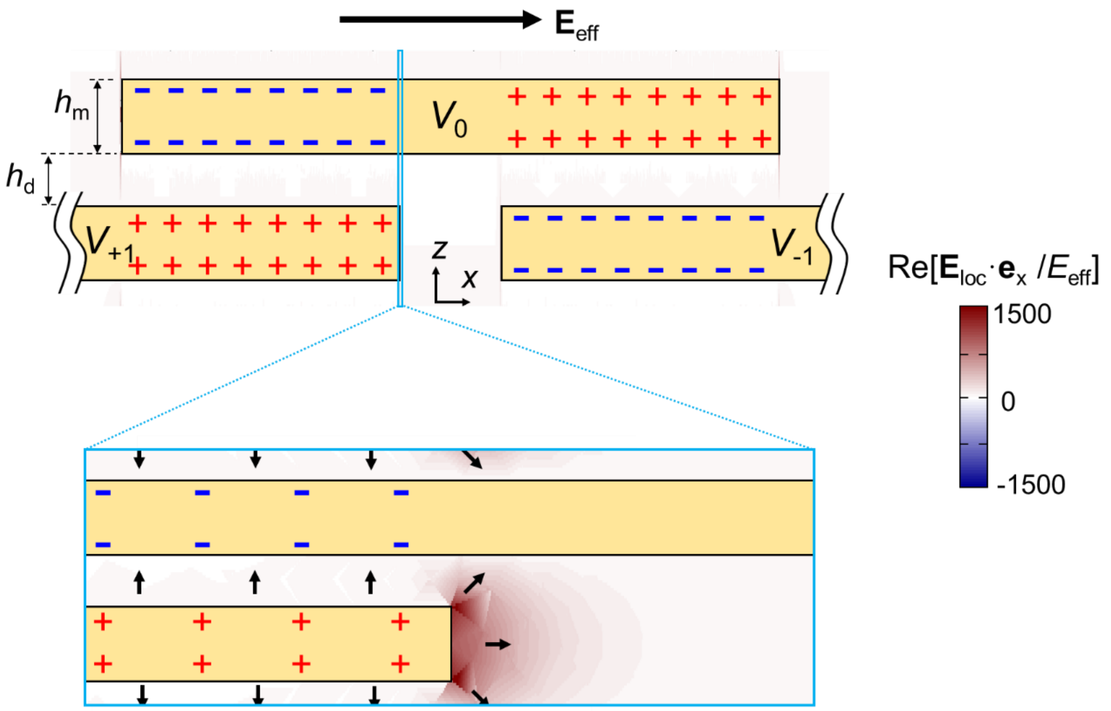

Supplementary Figure 3 | The *x*-directional local electric field profile The *x*-directional local electric field is plotted for the structure in Fig. 1c. Unlike *z*-directional local electric field in Fig. 1c, *x*-directional local electric field are almost negligible except near the tip of the metal strip even though macroscopic electric field is in *x*-direction. As h_d, h_m << *a*, the *z*-axis of the upper figure is exaggerated while the magnified figure at the bottom is drawn to scale. The black arrows provide visual guides for local electric field directions.

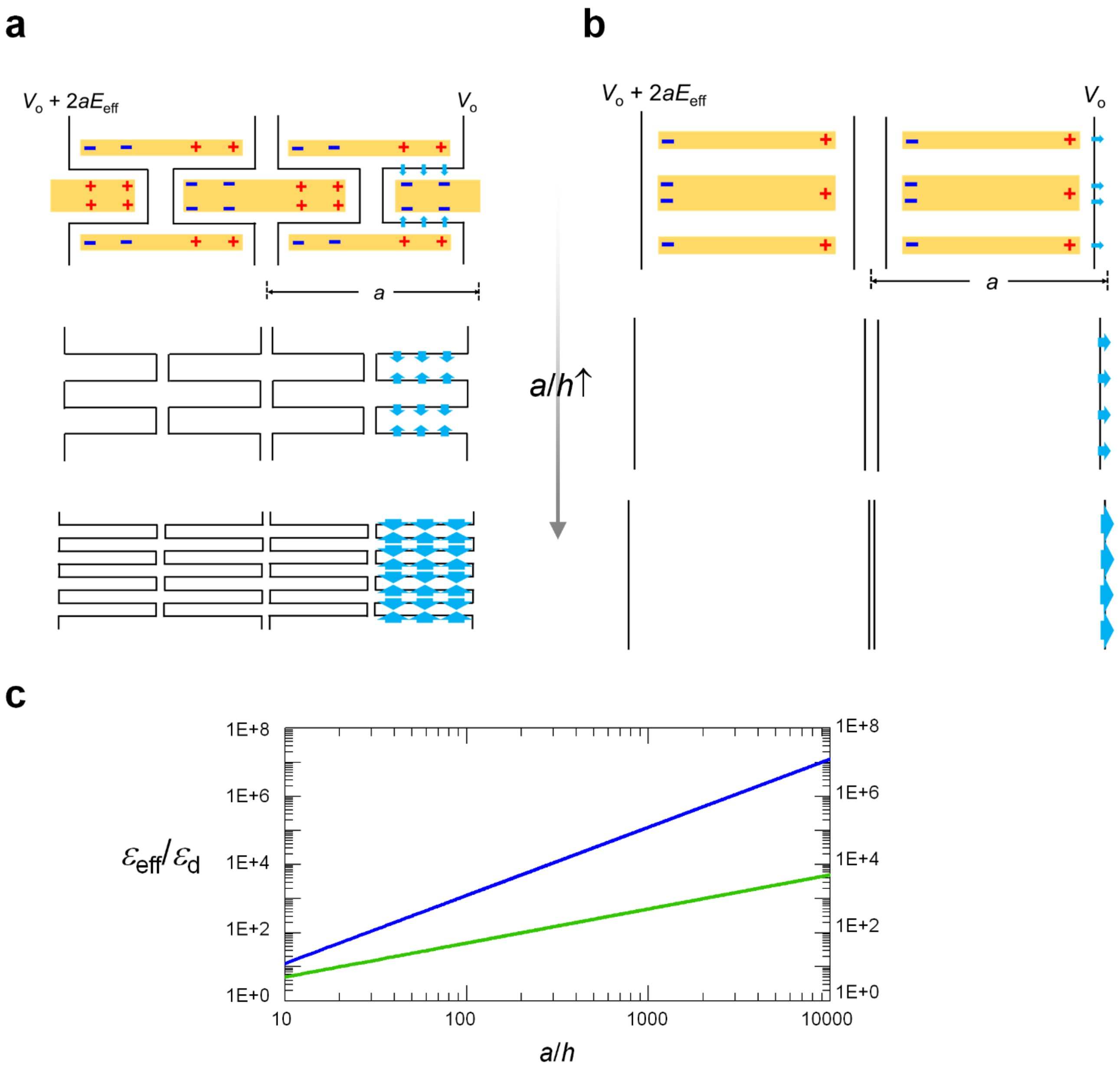

Supplementary Figure 4 | Equivalent space-filling curve for the mesoscopic crystal (a) Equipotential curves of the proposed structure for various aspect ratio. The curve follow the dielectric host between metals. As the aspect ratio, a/h, increases, the total length of the equipotential curve within a unit cell remains almost constant, and the local dipole moment (blue arrow) per unit length of the space-filling curve increases linearly (graphically indicated by thicker blue arrows). It is assumed that $h_d = h_m = h$. (b) Corresponding curves of a straightly aligned metal plate structure for various aspect ratios. Although the dipole moment density along the curve increases, the total length of the curve in a unit cell decreases as h decreases (because it is just $2h$). In this case, the curve occupies infinitesimally small region of the space

in the limit. (c) The dielectric constant enhancement factor for both structures as a function of the aspect ratio. The space-filling mesoscopic crystal and the straightly aligned plate array exhibit a quadratic (blue line) and a linear (green line) dependence on the aspect ratio.

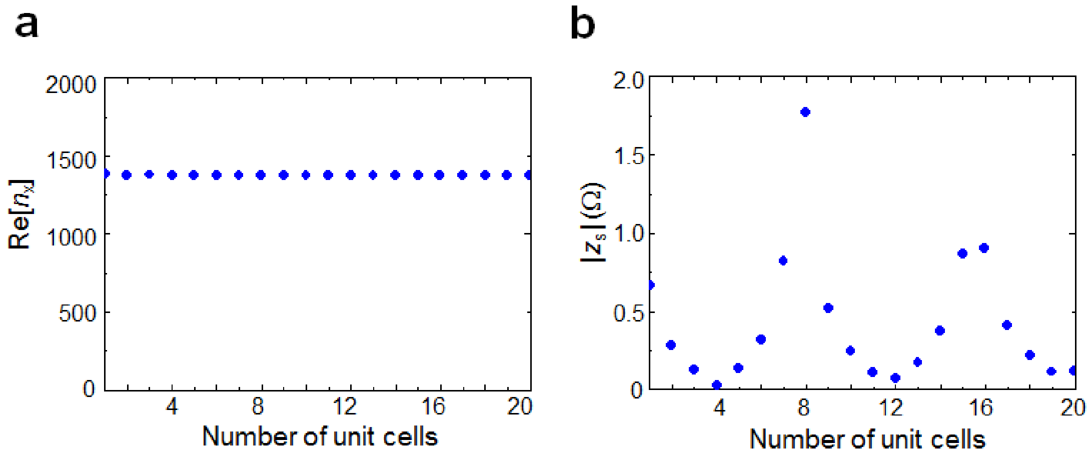

Supplementary Figure 5 | Effective optical property as a function of the crystal thickness The simulation setup and the structural parameters were same as Fig. 2. Here, we assume biaxial structure. The number of unit cells in the vertical direction was varied from 1 to 20 to investigate electromagnetic properties of the proposed structure as a function of the crystal thickness. (a) The real part of effective refractive index is plotted. Thickness independent effective refractive index implies that the proposed structure is well explained by a bulk homogenized medium. (b) Magnitude of the retrieved surface impedance is plotted. Fabry-Perot resonance is clearly visible when optical thickness of the crystal becomes integer multiples of a half wavelength, as expected for a finite thickness slab made of a homogeneous bulk material.

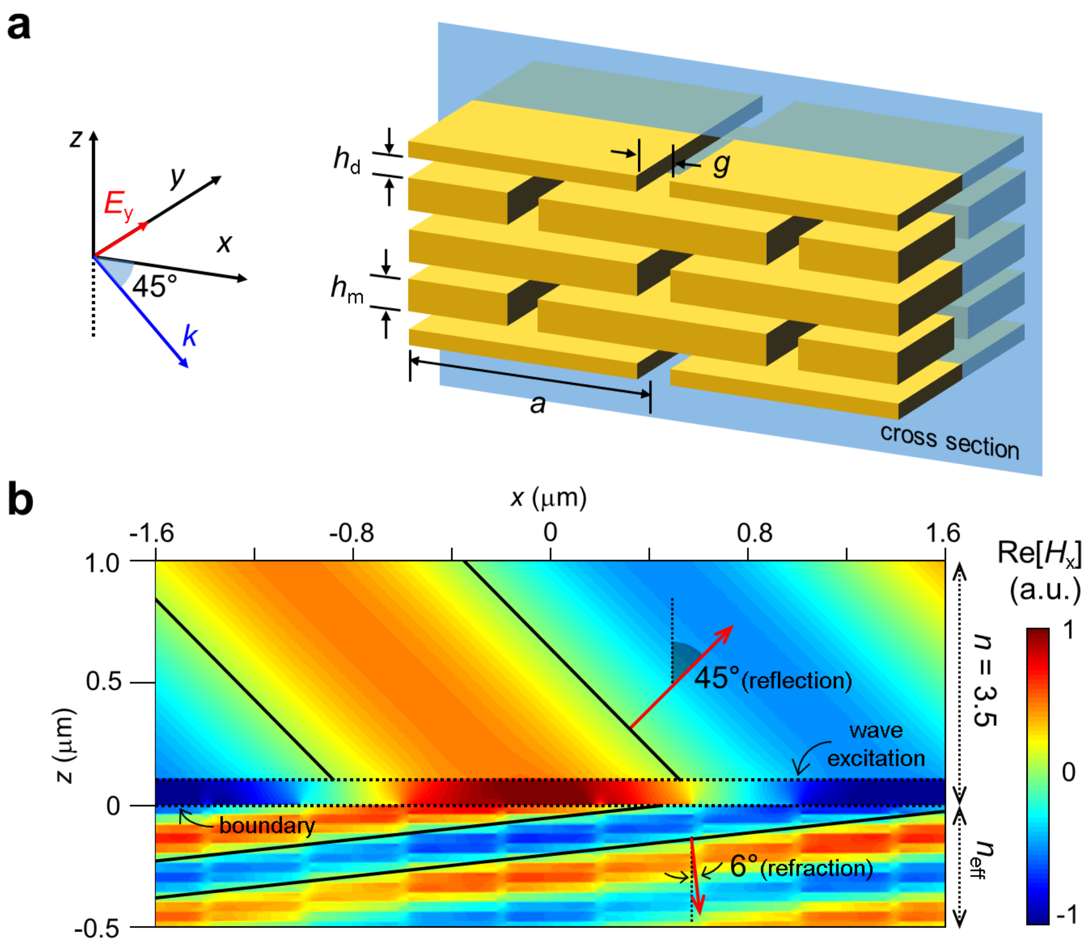

Supplementary Figure 6 | Snell's law for the mesoscopic crystal (a) The three-dimensional numerical simulation setup is shown. The transverse electric (TE) wave of 7.5 μm free space wavelength is incident on the mesoscopic crystal with 45 degree angle of incident. The wavevector is in the *x*-*o*-*z* plane. The refractive index of upper semi-infinite region is set to 3.5. The remaining region is assumed to be filled with the mesoscopic crystal (a = 400 nm, g = 40 nm, $h_m = h_d$ = 10 nm). The lossy Drude model gold (plasma frequency: 2.18 PHz, collision frequency: 6.45THz) and a dielectric with refractive index 1.4 was used for the mesoscopic crystal in this simulation. (b) Re[H_x] recorded on the cross section in (a). Black solid line indicate the wavefronts. Since the wavefronts are clearly visible inside the mesoscopic crystal as well, the Snell's law can be visually confirmed. The effective index of 24 calculated from the refraction angle (6°) and this value is consistent with analytically calculated value.

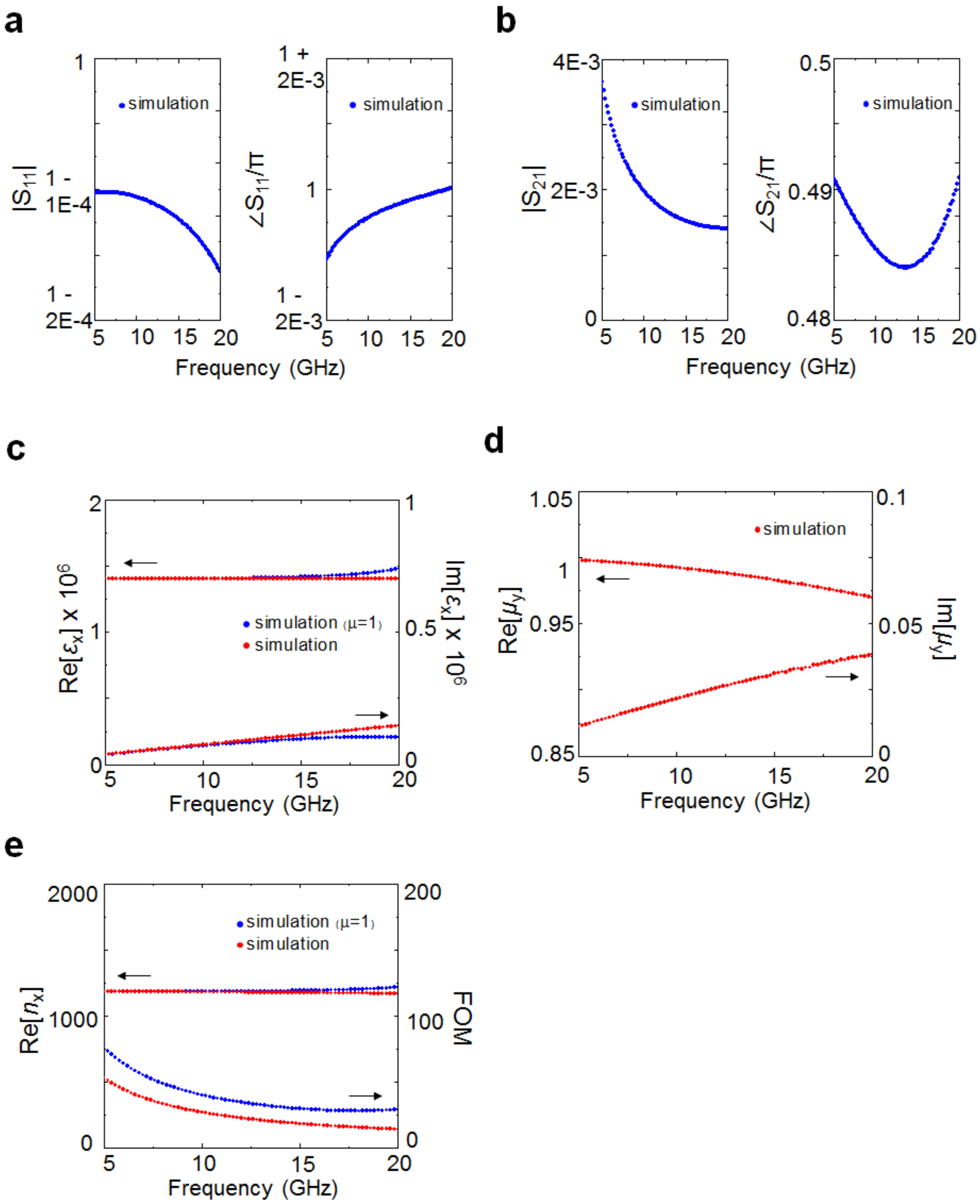

Supplementary Figure 7 | Relative permittivity and permeability from simulations Unit cell parameters are the same as in Fig. 2. (a) Magnitude and phase of the reflection coefficient, S_{11}. For (b)-(d), we assume uniaxial structure (Supplementary Note 2). (b) Magnitude and phase of the transmission coefficient, S_{21}. (c) Retrieved complex relative permittivity only from S_{21} assuming non-magnetic material (blue), and from both S_{11} and S_{21} without any assumption (red).

Two results are almost identical within the X-band frequency range (8.5–12 GHz). (d) Retrieved complex relative permeability. Retrieved permeability is close to vacuum permeability (within 5% difference), giving support for the non-magnetic assumption in Fig. 2 and 3, at least for the X-band. For higher frequencies, the thickness of the metal plates (400 nm) becomes comparable to the skin depth, thus the effective permeability deviates from the vacuum permeability. (e) Retrieved refractive index and FOM (Re[$n_{x(y)}$]/Im[$n_{x(y)}$]) only from S_{21} (blue), and from both S_{11} and S_{21} (red).

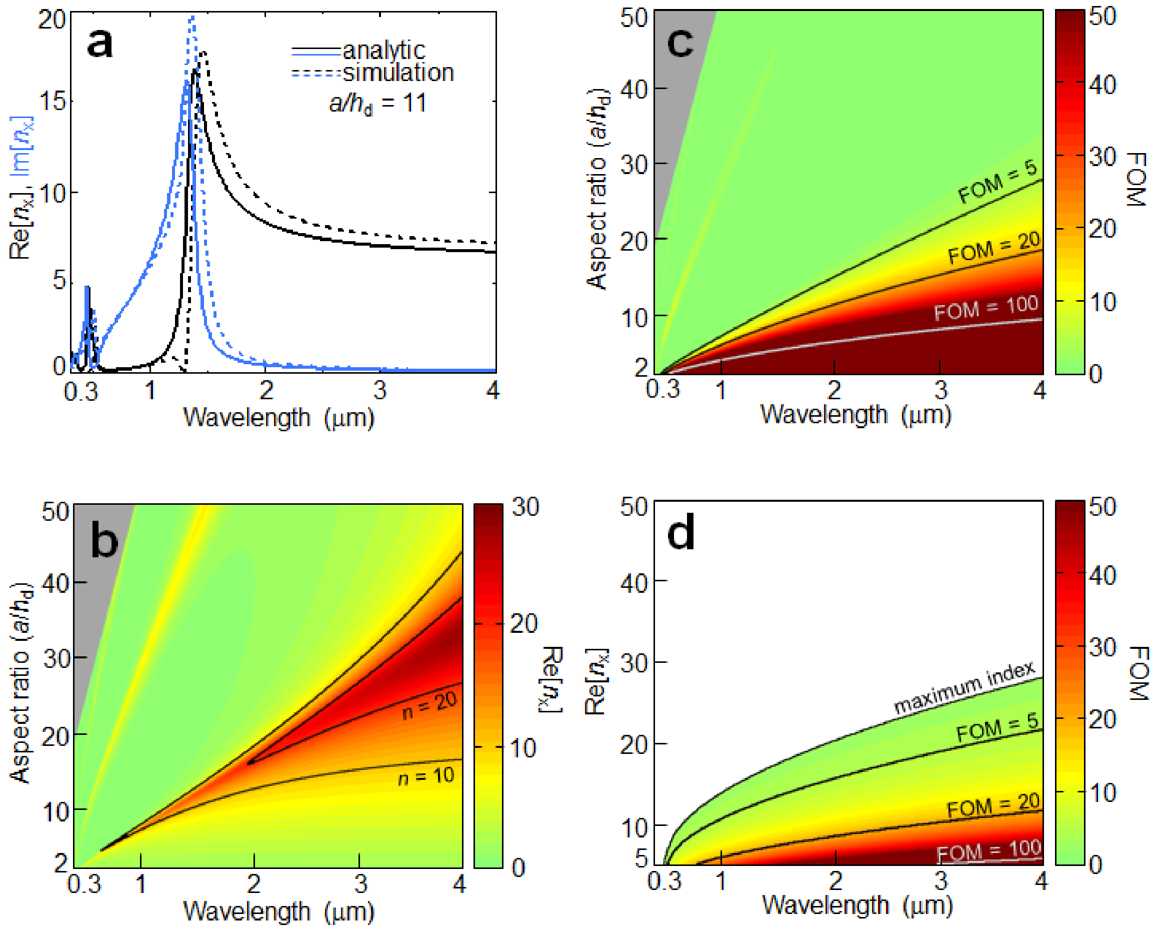

Supplementary Figure 8 | Recalculation of Fig. 4 with known parameters of silver Known material parameters for silver[16] were used for analytic calculation and simulation. (a) Similar with Fig. 4a, analytic calculation and retrieved refractive index from FDTD simulation show good agreement for the wavelength from 0.3 to 4 μm. (Black and blue color indicate real and imaginary part; Solid line and dashed line indicate analytic calculation and simulation, respectively.) (b)–(c) Wavelength dependent effective refractive index and FOM ($Re[n_x]/Im[n_x]$) for various aspect ratio are showed. (d) Achievable range of effective index is plotted as a function of wavlength. The effective index can be around 8 with FOM of 5 at wavelength of 600nm.

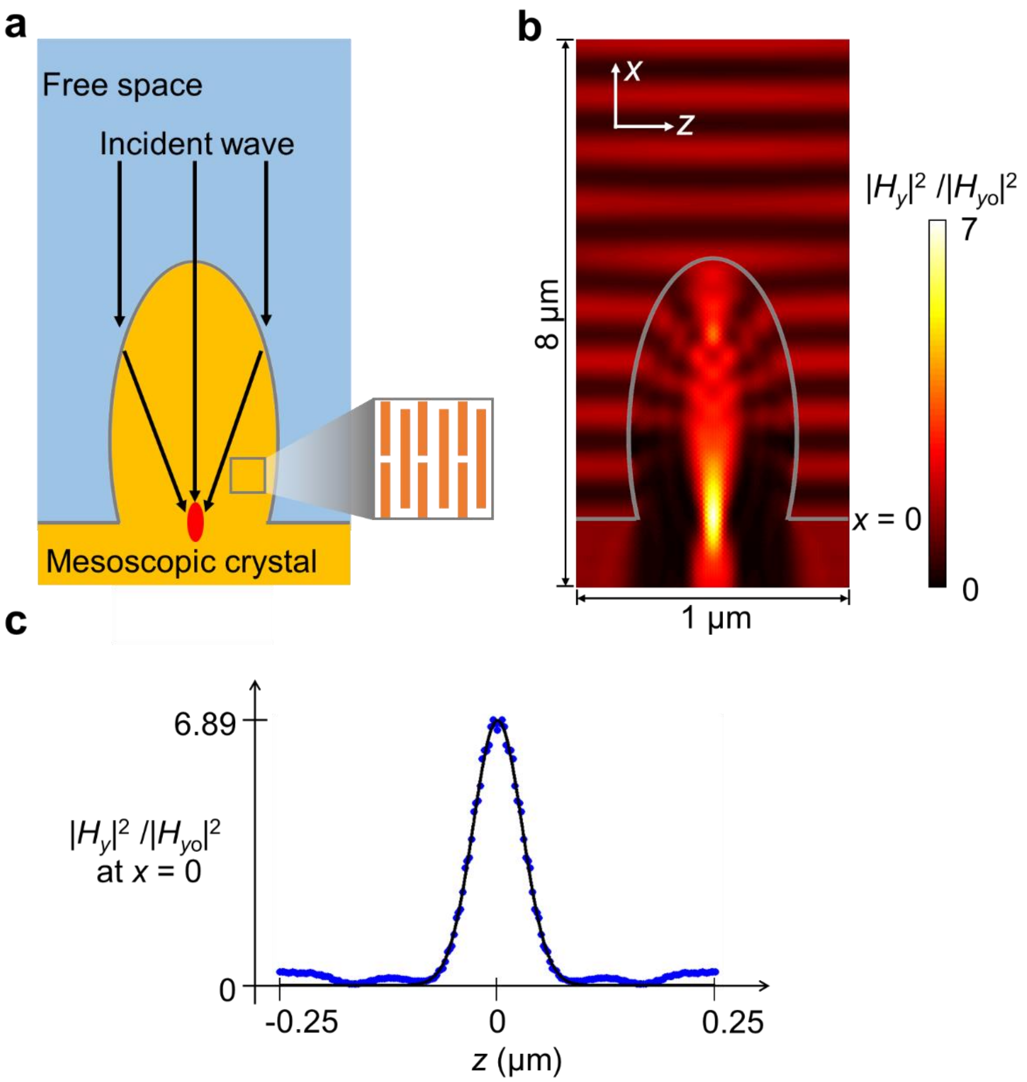

Supplementary Figure 9 | Sub-wavelength focusing with the mesoscopic crystal lens (a) A schematic of a convex lens made of the proposed mesoscopic crystal for subwavelength focusing. (b) Magnetic field intensity profile normalized by the incident magnetic field intensity, from a two-dimensional numerical simulation. The boundary between the mesoscopic crystal and free space is indicated with a grey solid line. The incident plane wave with free space wavelength, λ_o, of 1.55 μm was focused at the bottom of the mesoscopic crystal lens ($x = 0$). From the analytic model, the effective refractive index of the mesoscopic crystal is $n_x \approx 14.7$, $n_z \approx 2$ at $\lambda_o = 1.55$ μm. (b) Normalized magnetic field intensity at the focal plane (blue dots), and a Gaussian fitting (black solid line) with FWHM = 0.0657 μm, which is $\lambda_o/23.6$. Known complex permittivities of aluminum were used and a dielectric with index 1.4 were assumed. The structural dimensions were $g = h_d = h_m = 5$ nm, $a = 80$ nm.

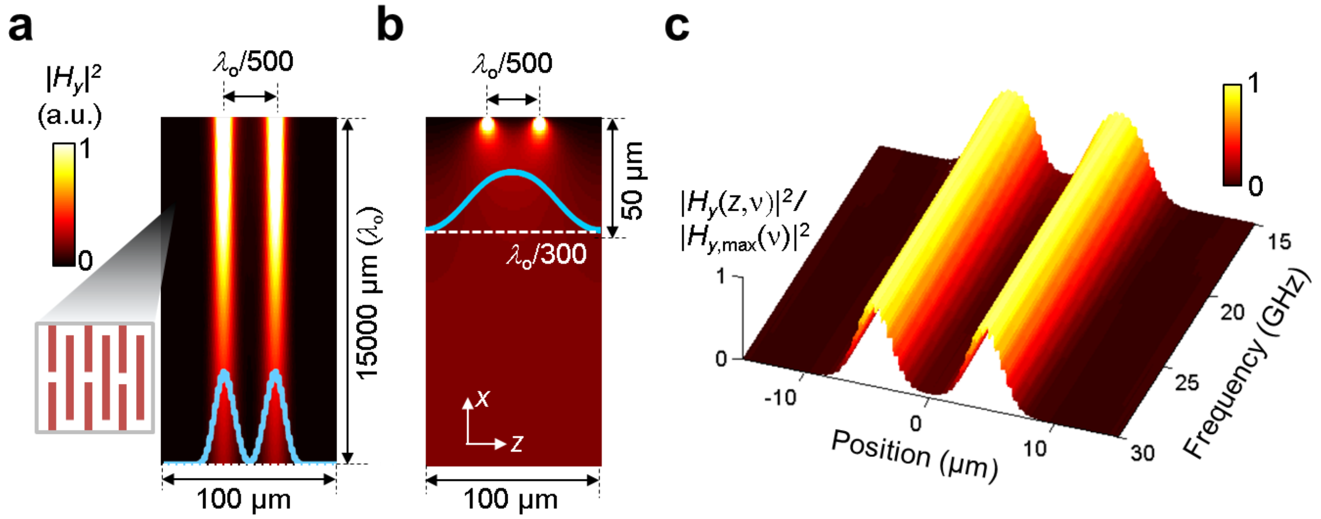

Supplementary Figure 10 | Deep sub-wavelength image transfer Magnetic field intensity (a.u.) is shown at 20 GHz. (a) Two adjacent (λ_o/500) in-phase dipoles were observed well-separated at 1λ_o, where λ_o = 15 mm. (b) In contrast, a uniform dielectric (SiO_2) medium could not transfer the separated image over a few tens of micrometers. The bright blue solid curves are the line plots of the magnetic field intensity after 1λ_o (a) and λ_o/300 (b) propagation, respectively. For the mesoscopic crystal, the structural dimension were a = 1200 µm, g = 40 µm, h_m = h_d = 400 nm and materials were same as the simulation in Fig. 2. (c) Microwave broadband sub-wavelength imaging performance of the mesoscopic crystal for 15–30 GHz is demonstrated by plotting the magnetic field intensity normalized by its maximum value at each frequency after 15 mm propagation, for two in-phase dipole sources separated by 30 µm. Similar image transfer performance as in (a) is maintained over the entire frequency range.

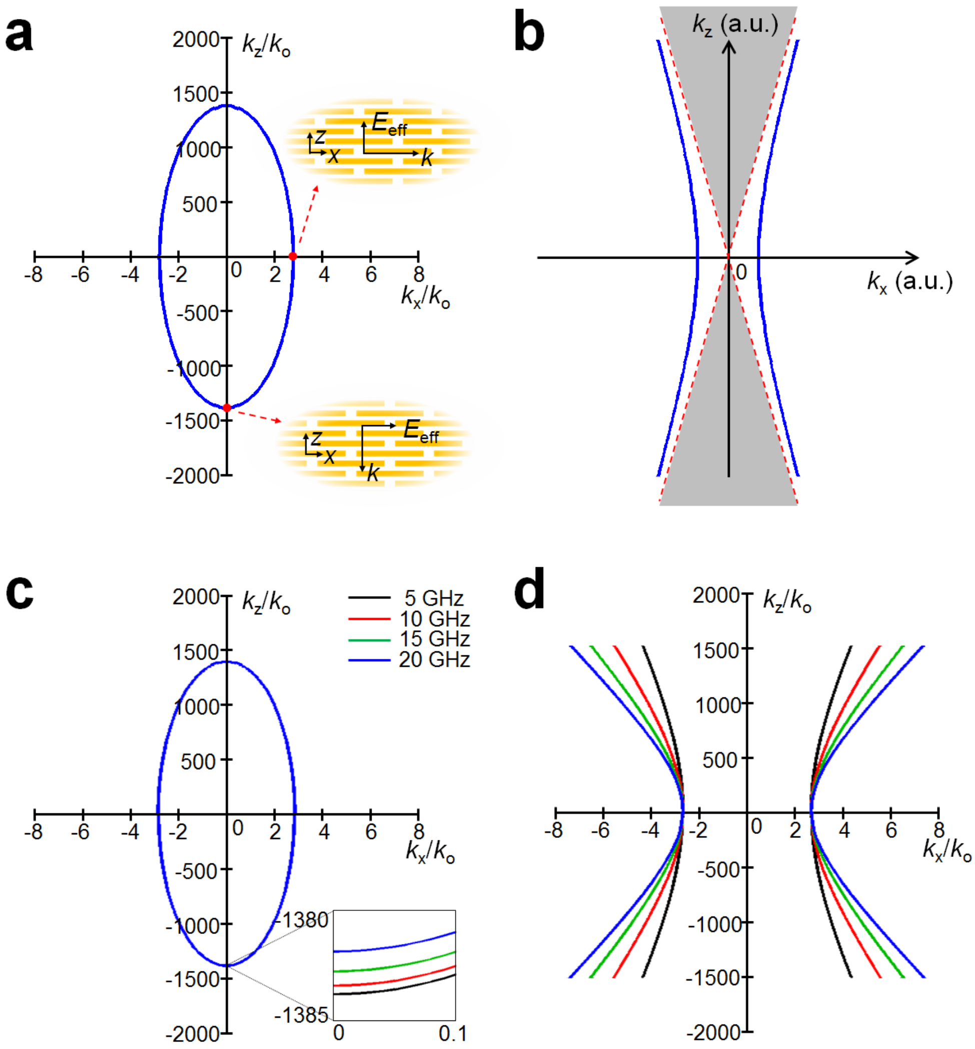

Supplementary Figure 11 | Two-dimensional Equi-Frequency Contour (EFC) (a) Calculated 2-dimensional EFC of the proposed mesoscopic crystal (biaxial case) at 10 GHz for transverse magnetic (TM) wave (magnetic field in *y* direction). The EFC is elliptic in shape, with extreme anisotropy. The two red dots indicate the wave propagating in the *x* and *z* directions. The structural dimensions were assumed to be the same as those shown in Fig. 2. The magnitude of the wavevector is normalized by that of vacuum. (b) Two-dimensional EFC of general hyperbolic media. There exists a range of directions in which electromagnetic waves cannot propagate (shaded region) between the two asymptotes (red dashed lines) of the hyperbola. (c) Normalized EFCs of the proposed structure for various frequencies. Black, red, green, and blue curves correspond to 5, 10, 15, and 20 GHz, respectively. The EFC shows almost no dispersion. (d) Normalized EFC of the hyperbolic medium (wire structure) shows significant dispersion. Colors correspond to the same frequencies as those in (c). To calculate

the effective permittivity of the wire medium, we assume that the wire thickness is smaller than skin depth (irrotational electric field approximation), and that the metal volume fraction is 1 % of the total volume. The longitudinal effective permittivity values were calculated using the weighted arithmetic mean of the filling matrix (SiO_2) and the metal (copper) permittivities. The transverse effective permittivity was calculated using the weighted harmonic mean.

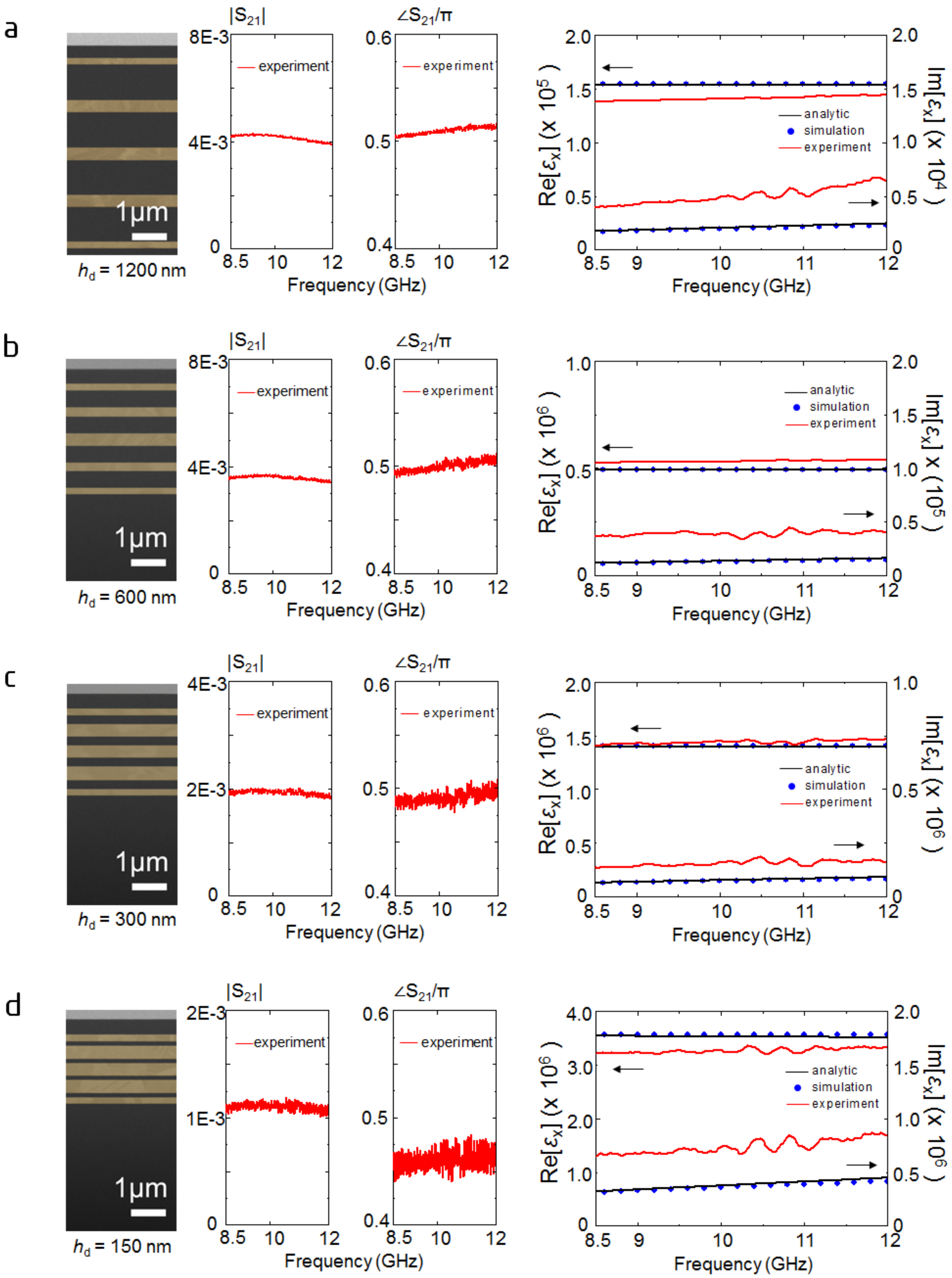

Supplementary Figure 12 | S_{21} raw data and homogenized dielectric constants Broadband

measurement was conducted for samples with different dielectric thickness. S_{21} values shown here are raw data, before applying the moving average. For the retrieval of the effective dielectric constant, moving averaged S_{21} values were used. Dielectric thicknesses are (a) h_d = 1200 nm, (b) h_d = 600 nm, (c) h_d = 300 nm, and (d) h_d = 150 nm. All other unit cell parameters are the same as in Fig. 2.

Supplementary Note 1 | Explanation of effective electric permittivity with PEC using capacitance formalism

In the following, we show that the effective AC dielectric constant of the proposed crystal (Fig. 1a) can become gigantic when h_d, $h_m << a$. We conduct a simple thought experiment involving a parallel plate capacitor, whose capacitance is well-known as $C = \varepsilon_0\varepsilon A/d$, where ε_0, ε, A, and d are the vacuum permittivity, the dielectric constant of the filling dielectric, the area of one face of the plates, and the distance between the plates, respectively. The above expression assumes that electric fields are confined to the region between two plates and fringing fields in the outside region can be ignored, which is valid if the widths of plates are much larger than d. The expression, originally derived for static voltages, remains valid for time-varying voltages if the involved frequency is sufficiently low such that the wavelength is much larger than the capacitor dimensions (i.e., the phase-retardation effect is negligible in the length scale involved). As the capacitance is directly proportional to the dielectric constant, one can measure the AC dielectric constant of an unknown material by filling a parallel plate capacitor of a known dimension with the material and measuring the capacitance.

We imagine a capacitor whose plates are many times larger than a but still much smaller than the wavelength. The distance between plates is exactly $a/2$ and we insert a biaxial version of our proposed crystal (Fig. 1c, Supplementary Fig. 2b) into the capacitor as shown in Supplementary Fig. 1a as it simplifies the problem to an effectively 2-dimensional one as in explanation of effective dielectric constant in main text. The result can be converted to the uniaxial effective dielectric constant by considering proper geometric factor (Supplementary Note 2). The use of a half unit cell in x-direction results in the same conclusion as the use of an integer multiples of a unit cell due to the mirror symmetry of the crystal. From the geometry, it is apparent that the metal plates of our crystal has an electric potential either V or 0, depending on which capacitor plate they are attached to. One can draw an equipotential curve of $V/2$ (the red dashed line in Fig. 1c) that follows the center of the dielectric spacer region. In the limit h_d, $h_m \rightarrow 0$, this equipotential curve becomes the space-filling curve that fills the entire space within the capacitor and the total length of the curve diverges to infinity. Locally, the electric field within the dielectric spacer region is perpendicular to this equipotential curve with magnitude V/h_d, just as in a parallel plate capacitor with plates h_d apart from each other. From the Gauss's law, the surface charge density associated with this electric field is $\varepsilon_0\varepsilon_d V/h_d$, where ε_d is the dielectric constant of the dielectric spacer. Thus, the total charge (per unit length in y-

direction) is this surface charge density times the length of the equipotential curve, which diverges in the thin spacer limit. For non-zero h_d and h_m, the total charge is $(\varepsilon_0\varepsilon_d V/h_d)\cdot[aL_z/2(h_d+h_m)]L_y$, where L_y and L_z are the width of the capacitor plates in y- and z-directions. Hence, $C = \varepsilon_0\varepsilon_d a L_y L_z/[2h_d(h_d+h_m)]$. Comparing this with the capacitance expression in the beginning of this argument, we find homogenized (effective) dielectric constant, $\varepsilon_{eff} = \varepsilon_d a^2/[4h_d(h_d+h_m)]$ as in Supplementary Fig. 1b.

Supplementary Note 2 | Analytic model for the effective electric permittivity considering the finite metal permittivity

A rigorous analytic model of the effective permittivity can be derived considering the finite and complex electric permittivities of the metal and the filling dielectric. The lateral dimension of unit cell is denoted as a, the thickness and the permittivity of metal plates and dielectric are (h_{m}, ϵ_{m}) and (h_{d}, ϵ_{d}), respectively. The electric field inside the vertical dielectric gap is $E_{\mathrm{zd}}(x)$ and the horizontal component of electric field inside the metal is $E_{\mathrm{xm}}(x)$. Both quantities are assumed to be constant in the vertical direction within each region due to the small scale of h_{m} and h_{d}. In the quasi-static, source-free condition, $\nabla \times \mathbf{E} = 0$ and $\nabla \cdot \mathbf{D} = 0$. The irrotational nature of E field dictates that the integration of E field along an arbitrary path from the center (x = 0, z = 0) of one plate (plate 1) to the center (x = a/2, z = h_{m}+h_{d}) of another plate (plate 2) should be invariant, while the solenoidal nature of D field relates D_x and D_z fields (Supplementary Fig. 2a). Thus,

$$\begin{cases} \int_0^{x'} E_{x\mathrm{m}}(x, \text{plate 1})\,dx + h_{\mathrm{d}} E_{z\mathrm{d}}(x') + \int_{x'}^{\frac{a}{2}} E_{x\mathrm{m}}(x, \text{plate 2})\,dx = \text{constant} \\ \text{and} \\ \epsilon_{\mathrm{m}} \frac{\mathrm{d}E_{x\mathrm{m}}(x,\text{plate 1})}{\mathrm{d}x} \cdot \frac{h_{\mathrm{m}}}{2} = \epsilon_{\mathrm{d}} E_{z\mathrm{d}}(x). \end{cases} \quad (1)$$

Using the translational symmetry, $E_{x\mathrm{m}}(x, \text{plate 2}) = E_{x\mathrm{m}}(x - a/2, \text{plate 1})$ and the two-fold rotational symmetry around (x = a/4, z = (h_{d} + h_{m})/2) point, we obtain the expression for E_{zd} field which is proportional to the x-directional E field on the dielectric side of the interface between the metal plate and the lateral dielectric gap ($E_{x\mathrm{do}}$):

$$E_{z\mathrm{d}}(x) = E_{x\mathrm{do}} \frac{g}{h_{\mathrm{d}}} \left(1 - \frac{\epsilon_{\mathrm{d}}}{\epsilon_{\mathrm{m}}}\right) \frac{\exp\left[P\left(x+\frac{a}{4}\right)\right] + \exp\left[-P\left(x+\frac{a}{4}\right)\right]}{(2+Pg)\exp[PL] + (2-Pg)\exp[-PL]}\ , \quad (2)$$

where the new parameters are $P = \sqrt{\frac{4\epsilon_\mathrm{d}}{\epsilon_\mathrm{m} h_\mathrm{m} h_\mathrm{d}}}$ and $L = \frac{a}{4} - \frac{g}{2}$. Similarly, $E_x(x)$ can also be calculated. Effective epsilon is estimated by considering average electric field and average electric displacement inside the unit cell as explained in the main text,

$$\epsilon_\mathrm{eff} = \frac{(\epsilon_\mathrm{m} h_\mathrm{m} + \epsilon_\mathrm{d} h_\mathrm{d}) Q \left(1 - \frac{\epsilon_\mathrm{d}}{\epsilon_\mathrm{m}}\right) + 2\epsilon_\mathrm{d} h_\mathrm{m} + \epsilon_\mathrm{d} h_\mathrm{d} + \frac{\epsilon_\mathrm{d}^2}{\epsilon_\mathrm{m}} h_\mathrm{d}}{2(h_\mathrm{m} + h_\mathrm{d})\left\{\frac{1}{2}\left(1 - \frac{\epsilon_\mathrm{d}}{\epsilon_\mathrm{m}}\right) Q + \left(\frac{\epsilon_\mathrm{d}}{\epsilon_\mathrm{m}} - \frac{g}{a} \frac{\epsilon_\mathrm{d}}{\epsilon_\mathrm{m}} + \frac{g}{a}\right)\right\}}, \tag{3}$$

$$\text{where } Q = \frac{Pg\ \{\exp(PL) - \exp(-PL)\}}{(2+Pg)\exp(PL) + (2-Pg)\exp(-PL)}.$$

When $\epsilon_\mathrm{m} \to \infty$, $'\epsilon_\mathrm{m} Q'$ converge to $\frac{2\epsilon_\mathrm{d} g L}{h_\mathrm{m} h_\mathrm{d}}$. Then, $\epsilon_\mathrm{eff} \to \epsilon_\mathrm{d} \left[\frac{a^2}{4(h_\mathrm{m}+h_\mathrm{d})h_\mathrm{d}} - \frac{ag}{2(h_\mathrm{m}+h_\mathrm{d})h_\mathrm{d}} + \frac{h_\mathrm{m} a}{(h_\mathrm{m}+h_\mathrm{d})g} + \frac{h_\mathrm{d} a}{2(h_\mathrm{m}+h_\mathrm{d})g}\right] \approx \epsilon_\mathrm{d} \left[\frac{a^2}{4(h_\mathrm{m}+h_\mathrm{d})h_\mathrm{d}}\right]$, for $h_\mathrm{m}, h_\mathrm{d} \ll g \ll a$. Therefore, the general result is reduced to the simple PEC model described in the main text. For the uniaxial structure, the overlapped region reduced to $\frac{a-2g}{a}$ compared with biaxial structure (Supplementary Fig. 2b, 2c). Therefore, effective permittivity of uniaxial alternatively arranged metal plates become approximately, $\epsilon_\mathrm{u} \approx \epsilon_\mathrm{b}\left(\frac{a-2g}{a}\right)$. Uniaxial relative effective permittivity, $\frac{\epsilon_\mathrm{u}}{\epsilon_\mathrm{o}}$ was used to compare with experiment and simulation in Figs. 2 and 3 in the main text and Supplementary Figs. 7 and 12.

Supplementary Note 3 | Transfer matrix method for retrieval of effective electromagnetic parameters

For the experimental data in Figs. 2 and 3, the effective refractive index was retrieved by the conventional transfer matrix method. Since the reflection coefficient S_{11} is very close to 1 in magnitude and, hence, a small measurement error in S_{11} results in large deviation in the extracted parameters. Hence, S_{21} was used alone, and the effective permeability was assumed to be the vacuum permeability. The S_{21} was measured by a vector network analyzer (8510c, Agilent) with X-band waveguides (X281C, Agilent).The incident guided mode in X-band rectangular waveguide was considered as TE_{10} mode. Therefore, the material response to electromagnetic wave only depends on ε_{xx}, μ_{yy}, μ_{zz} where the coordinate defined as Supplementary Fig. 2. Since we assumed μ (= μ_{yy} = μ_{zz}) = 1, the fabricated mesoscopic crystal can be treated as effective isotropic medium with $\varepsilon_{\mathrm{iso}} = \varepsilon_{xx}$, $\mu_{\mathrm{iso}} = 1$. The refractive index can be calculated as $n = [\varepsilon_{\mathrm{iso}}\cdot\mu_{\mathrm{iso}}]^{1/2}$ (noted as $n_{x(y)}$ in the manuscript). Then, the transfer matrix of the mesoscopic crystal becomes that of simple isotropic slab with oblique plane wave incidence. The overall transfer matrix become,

$$\begin{bmatrix} A \\ B \end{bmatrix} = \begin{bmatrix} \text{transfer matrix} \\ \text{of} \\ \text{protective layer} \end{bmatrix} \begin{bmatrix} \text{transfer matrix} \\ \text{of} \\ \text{the mesoscopic} \\ \text{crystal} \end{bmatrix} \begin{bmatrix} \text{transfer matrix} \\ \text{of} \\ \text{substrate} \end{bmatrix} \begin{bmatrix} C \\ D \end{bmatrix}, \qquad (4)$$

$$\text{where,} \quad S_{21} = \frac{C}{A}.$$

The protective layer is 500 nm-thick, RF-sputtered SiO_2 and the substrate is 0.5 mm-thick, diced quartz wafer. The effective parameters were retrieved by numerically calculating transfer matrix components of the mesoscopic crystal with oblique incident TE plane waves.

For the numerical data in Figs. 2 and 3, there was no protection layer and the substrate, and the incident wave was normally incident plane waves. Therefore, the S_{21} have a simpler form,

$$S_{21} = \frac{1}{\cos(n_{\text{eff}}k_0 d) - \mathrm{i}(\frac{z_0^2 + z_{\text{eff}}{}^2}{2z_0 z_{\text{eff}}})\sin(n_{\text{eff}}k_0 d)} \ , \tag{5}$$

where

z_0: wave impedance of vacuum

$z_{\text{eff}} = \dfrac{z_0}{n_{\text{eff}}}$: effective wave impedance of the mesoscopic crystal

n_{eff}: effective refractive index of the mesoscopic crystal

k_0: magnitude of wave vector of incident wave in vacuum

d: depth of the mesoscopic crystal.

The effective permeability was also assumed to be the vacuum permeability for direct comparison with experimental results.

Additionally, the effective permeability was also retrieved in Supplementary Fig. 7 by utilizing S_{11}, which is

$$S_{11} = \frac{\mathrm{i}(\frac{z_1^2 - z_{\text{eff}}{}^2}{2z_1 z_{\text{eff}}})\sin(n_{\text{eff}}k_0 d)}{\cos(n_{\text{eff}}k_0 d) - \mathrm{i}(\frac{z_0^2 + z_{\text{eff}}{}^2}{2z_0 z_{\text{eff}}})\sin(n_{\text{eff}}k_0 d)} \ . \tag{6}$$

Supplementary Note 4 | Copper permittivity from measured dc conductivity

Since X-band frequency (8.2– 12.4 GHz) is much smaller than collision frequency of copper (few THz), the imaginary part of the permittivity of copper is much larger than its real part and the permittivity can be calculated from measured dc conductivity as following,

$$\epsilon \approx \mathrm{i}\epsilon'' = \frac{\mathrm{i}\sigma_{\mathrm{dc}}}{\epsilon_0\omega} \ . \tag{7}$$

The measured sheet resistance of 400 nm thickness copper film was $0.0473\ \Omega$ (standard deviation: 1.01×10^{-3}), and corresponding dc conductivity is $5.288 \times 10^{7}\ \Omega^{-1}\mathrm{m}^{-1}$. Therefore, relative permittivity value of copper, $\epsilon = \mathrm{i}\frac{5.973\times10^{18}}{\omega}$ was used in the analytic model, simulations, and experiments.
